# METTL3-associated YTHDF1-COX-1 axis is linked to reduced antiplatelet response to aspirin in older adults

**DOI:** 10.1515/biol-2025-1375

**Published:** 2026-11-06

**Authors:** Jiaqi Zhang, Wenwen Liu, Xiting Wang, Xiahuan Chen, Meilin Liu

**Affiliations:** Department of Geriatrics, Peking University First Hospital, Beijing, 100034, China

**Keywords:** aspirin, platelet aggregation, RNA m6A methylation, methyltransferase-like 3, YTH N6-methyladenosine RNA binding protein 1

## Abstract

This study explores the role and mechanism of RNA m6A methylation in reduced antiplatelet response to aspirin in older adults. A total of 34 patients aged ≥60 years routinely receiving 100 mg/d aspirin were enrolled. Based on light transmission aggregometry (LTA), subjects were divided into a low-response group (*n* = 14, platelet aggregation rate (PAR) ≥12 %) and a high-response group (*n* = 20, PAR ≤7 %). Patients with a low aspirin response exhibited higher levels of m6A, accompanied by elevated methyltransferase-like 3 (METTL3) and YTH N6-methyladenosine RNA binding protein 1 (YTHDF1) (*p* < 0.05). Serum levels of prostaglandin H2 (PGH2) and thromboxane B2 (TXB2) were also elevated in the low-response group. Spearman correlation revealed that PAR and PGH2 were positively correlated with both METTL3 and YTHDF1 mRNA, while TXB2 positively correlated with METTL3 mRNA (*p* < 0.05), indicating the association of m6A degree with aspirin response. Mechanistically, METTL3 overexpression in MEG-01 cells enhanced megakaryocyte proliferation and upregulated *YTHDF1*, which subsequently increased the translation efficiency of cyclooxygenase 1 (COX-1) mRNA and elevated *COX-1* protein. RNA immunoprecipitation confirmed that *YTHDF1* directly bound to COX-1 mRNA in a METTL3-dependent manner. The METTL3/*YTHDF1-COX-1* axis may attenuate the antiplatelet efficiency of aspirin by modulating AA metabolism and megakaryocyte function, providing a potential biomarker and target for aspirin low-responsiveness.

## 1 Introduction

Cardiovascular disease is the predominant cause of mortality in older adults and the antiplatelet activities of aspirin provide utility for prevention and management of cardiovascular conditions [1], 2]. Aspirin has contributed to reduced cardiovascular/cerebrovascular events and mortality rates but has varying antiplatelet effects and impact on patient outcomes [3]. The identification of mechanistic explanations for inter-patient variation may allow aspirin effects to be optimized, improving the quality of life and long-term survival of elderly patients. Although aspirin remains a cornerstone antiplatelet agent for secondary prevention of atherothrombotic events, substantial inter-individual variability in aspirin responsiveness has been reported, particularly in older patients with cardiovascular disease [4]. This variability may be influenced by platelet turnover, inflammatory status, comorbidities, concomitant medications, and molecular regulators of arachidonic acid metabolism [5]. Recent studies in other disease contexts have also highlighted that immune-cell inflammatory imbalance may alter systemic inflammatory status, supporting the broader concept that immune and inflammatory regulation can influence disease-related cellular responses. However, direct evidence linking these findings to aspirin responsiveness remains limited.

Suboptimal suppression of platelet aggregation is often termed “aspirin low response” and it is a significant risk factor for thrombotic events [6]. However, aspirin low response lacks a clear definition and underlying mechanisms remain unclear [7]. Accumulating epidemiological data indicate that the prevalence of aspirin low responsiveness or resistance ranges from 5 % to 60 % among older adults, with this wide variation largely depending on the assay used to assess platelet function, such as light transmission aggregometry (LTA), VerifyNow, or thromboelastography [8], 9]. This high heterogeneity in older adults presents a major challenge for precise antiplatelet therapy. Aspirin is an irreversible inhibitor of cyclooxygenase 1 (*COX-1*, also known as prostaglandin endoperoxide synthase 1, *PTGS1*) and disrupts the conversion of arachidonic acid (AA) into the eicosanoid hormones that cause platelet aggregation (Figure S1) [10], 11]. Differential expression and posttranslational modification of enzyme and receptor genes, such as *COX-1*, cyclooxygenase 2, thromboxane A synthase 1 (*TBXAS1*), prostacyclin synthase (*PTGIS*) and prostacyclin receptor (*PTGIR*), on the pathway of AA metabolism have been linked to aspirin low response [12], [13], [14], [15], [16].

Over 100 types of posttranscriptional RNA modifications have been described, of which N6-methyladenosine (m6A) is the most abundant in eukaryotic cells. m6A methyltransferases, m6A demethylases and m6A reader proteins are involved in modification with m6A methyltransferases and demethylases controlling the dynamics and reader proteins affecting mRNA splicing, output, translation and degradation [17]. These processes have been implicated in various pathological mechanisms [18], 19]. Methyltransferase-like 3 (*METTL3*) is the only catalytic subunit in the methyltransferase complex and was the first component to be identified and isolated [20]. The m6A reader protein, YTH N6-methyladenosine RNA binding protein 1 (*YTHDF1*), interacts with the translation initiation complex to promote translation of m6A-modified mRNA [21]. Changes in *METTL3* expression lead to positive or negative regulation of reader protein synthesis, enabling changes to translation or degradation of target mRNAs [22]. m6A modifications have previously been linked to cardiovascular disease and *METTL3* expression implicated in myocardial hypertrophy, cardiac aging and atherosclerotic plaque progression [23], [24], [25]. *YTHDF1* expression has been correlated with angiogenesis and the generation and differentiation of hematopoietic stem cells [26], 27]. However, the impact of m6A modifications on AA metabolism, platelet generation and the antiplatelet effects of aspirin have been little studied. The current study aimed to explore the impact of m6A modification on the aspirin response and its underlying mechanisms.

## 2 Materials and methods

### 2.1 Patient selection and clinical data collection

The present study analyzed participants and biological samples from the Peking University First Hospital center of a multicenter, randomized, open-label, parallel-controlled aspirin trial (ChiCTR1800018517) [28]. A total of 34 patients aged ≥60 years received 100 mg/d aspirin as a primary or secondary prevention measure and were included. Inclusion criteria: 100 mg/d aspirin for at least 2 weeks; light transmission aggregometry (LTA) test producing AA-induced platelet aggregation rate (PAR) ≥12 % (low aspirin response) or ≤7 % (high aspirin response) after 2 weeks. The cutoffs used to define aspirin low response and high response were pre-specified based on the quartile distribution of platelet aggregation rates in our previous larger cohort, rather than being derived post hoc from the present study population. The parent study was approved by the Clinical Research Ethics Committee of Peking University First Hospital. Written informed consent was obtained from all participants and covered blood collection, storage, and subsequent molecular analyses related to aspirin responsiveness and platelet function. Exclusion criteria: aspirin allergy or contraindication, bleeding risk, use of other nonsteroidal anti-inflammatory drugs or steroids, concurrent administration of other antiplatelet agents, such as clopidogrel, ticlopidine or tirofiban, or anticoagulant drugs, such as warfarin, dabigatran or rivaroxaban.

**Informed consent:** Informed consent was obtained from all individuals participating in this study, or from their legal guardians or wards.

**Ethical approval:** The research related to human use has been complied with all the relevant national regulations, institutional policies and in accordance with the tenets of the Helsinki Declaration, and has been approved by the Clinical Research Ethics Committee of Peking University First Hospital on September 23, 2016 (Approval No. 2016[1175]).

### 2.2 PAR measurement and patient grouping

Following 2 weeks of aspirin intake, 2 mL blood samples were collected and AA-induced testing was performed using a 4-channel LBY-NJ4 LTA apparatus (PRECIL, Beijing, China). Centrifugation at 200×*g* for 10 min was conducted to prepare platelet-rich plasma (PRP) and centrifugation at 2,000×*g* for 10 min to prepare platelet-poor plasma (PPP). PRP was stimulated with 0.5 mg/mL AA and PAR was measured using LTA within 2 h of blood collection. PAR measurements were performed by the same team in the Department of Clinical Laboratory at Peking University First Hospital using standardized procedures; all team members had received standardized training.

AA-induced PAR reflects aspirin responsiveness. The aspirin low-response group was defined as PAR ≥12 % (upper quartile of the large-sample population) and the aspirin high-response group as PAR ≤7 % (lower quartile of the large-sample population) based on previously reported results and standardized for the current study [29]. All eligible participants at this center were included, comprising 14 with low aspirin responsiveness and 20 with high responsiveness.

### 2.3 RNA extraction and quantitative PCR (qPCR)

Total RNA was isolated from whole blood using a kit (Simgen, Hangzhou, China) and from cultured cells using RNA simple total RNA extraction kit (TIANGEN, Beijing, China) and reverse transcribed using a cDNA synthesis kit (Takara, Kyoto, Japan). The NCBI online tool, Primer-BLAST (www.ncbi.nlm.nih.gov/tools/primer-blast), was used to design primers for qPCR. The genes referred to are METTL3, METTL14 (methyltransferase-like 14), WTAP (Wilms tumor 1 associated protein), FTO (fat mass and obesity-associated protein), ALKBH5 (AlkB homolog 5, RNA demethylase), YTHDF1, YTHDF2 (YTH N6-methyladenosine RNA binding protein 2), PTGS1, PTGS2 (prostaglandin endoperoxide synthase 2), TBXAS1, PTGIS, PTGIR, CD41, and CD61. The primer sequences are provided in Table S1. Real-time qPCR was performed using a fast real-time PCR instrument (ABI 7500; Alameda, CA, USA) in a total reaction volume of 20 μL containing 10 μL 2 × SYBR Green Master Mix (Applied Biosystems, Foster City, CA, USA), 0.5 μL each of forward and reverse primers, 1 μL cDNA and 8 μL ddH_2_O. Relative mRNA expression was calculated using the 2^−ΔΔCq^ method. β-actin was used as the endogenous reference gene for whole-blood samples, whereas GAPDH was used for cultured MEG-01 cells.

### 2.4 Measurement of m6A

m6A modifications of total RNA were colorimetrically quantified using the m6A RNA methylation quantification kit (Abcam). Briefly, 200 ng total RNA was added per well with capture and detection antibody solution, according to the manufacturer’s instructions. Absorbance was measured at 450 nm using a microplate reader (Synergy H1; BioTek, Winooski, VT, USA).

### 2.5 Cell culture

The human megakaryoblastic leukemia cell line, MEG-01 (Procell, Wuhan, China), was cultured in RPMI-1640 (Gibco, Grand Island, NY, USA) supplemented with 10 % fetal bovine serum (FBS; Gibco) and 1 % streptomycin/penicillin (Sigma, St Louis, MO, USA) at 37 °C and 5 % CO_2_.

### 2.6 Enzyme-linked immunosorbent assay

PGH2 (Cloud-Clone Corp, Wuhan, China) and TXB2 (Abcam) were measured in human serum and cell culture supernatant using ELISA kits, according to the manufacturer’s instructions. Absorbance at 450 nm was measured using a microplate reader (Synergy H1; BioTek).

Because PGH2 is chemically unstable, serum and cell-culture supernatant samples were collected, processed, and stored according to the ELISA kit manufacturer’s instructions, and repeated freeze-thaw cycles were avoided before measurement.

### 2.7 Lentiviral infection

METTL3-overexpressing and METTL3 RNAi lentiviruses were purchased from Shanghai Jikai Gene Co., Ltd (Shanghai, China) and used to transfect MEG-01 cells following the manufacturer’s instructions. Cells were divided into 5 groups: normal control (Blank), METTL3-overexpressing (OE), overexpressing negative control (OE-NC), METTL3 knockdown (KD) and knockdown negative control (KD-NC). Cells were cultured as normal for 24 h and transfected at MOI of 100. Stable cell lines were screened using 1 μg/mL puromycin (Sigma) 48 h after transfection. Fluorescence was observed under a fluorescence microscope and transfection was verified by qPCR.

### 2.8 Flow cytometry analysis of cell surface antigens

MEG-01 cells were differentiated into mature megakaryocytes with phorbol 12-myristate 13-acetate (PMA, Sigma) at the concentrations and times indicated. Cells were incubated with FITC-conjugated CD41 and CD61 antibodies (Biolegend, San Diego, CA, USA) for 30 min at 25 °C, unbound antibody was washed away with cold phosphate-buffered saline (PBS; Gibco) and fluorescence-activated cell sorting flow cytometer (LSR Fortessa™ X-20, BD, Franklin Lakes, NJ, USA) was used with FlowJo V10.8.1 software (TreeStar Inc., Ashland, OR, USA) to analyze surface antigens. The gating strategy was as follows: debris was first excluded based on forward scatter area (FSC-A) and side scatter area (SSC-A), and the main MEG-01 cell population was selected. Where applicable, singlet events were further gated using FSC-A/FSC-H parameters. CD41- and CD61-positive populations were then defined using unstained controls or isotype controls to determine the positivity thresholds. The percentages of CD41-positive, CD61-positive, and CD41/CD61 double-positive cells were calculated within the gated viable cell population.

### 2.9 Protein extraction and western blot assays

Total protein was isolated with RIPA lysis buffer containing proteinase and phosphatase inhibitors (KeyGen Biotech, Nanjing, China) and concentration was determined with bicinchoninic acid assay (BCA; Beyotime Bio, Shanghai, China). Briefly, 20 μg of sample protein was separated by 4–20 % SDS-PAGE (Epizyme, Shanghai, China) and proteins were electrotransferred onto polyvinylidene fluoride membrane (PVDF; Millipore, Bedford, MA, USA). Membranes were blocked with a rapid blocking solution (Beyotime Bio) and incubated overnight at 4 °C with primary antibodies raised against METTL3, YTHDF1, YTHDF2, COX-1, COX-2 and TBXAS1 (Abcam) diluted 1:1,000 in dilution buffer (Beyotime Bio, Shanghai, China). Membranes were washed 3 times with Tris-buffered saline containing Tween (TBST; LABLEAD, Beijing, China), incubated with secondary HRP-conjugated goat anti-rabbit IgG (CST, Boston, MA, USA) diluted 1:1,000 in dilution buffer at 25 °C for 2 h, washed with TBST and proteins visualized (Thermo Fisher Scientific, MA, USA) and quantified with ImageJ software (ImageJ, NIH, Bethesda, MD, USA).

Primary antibodies against METTL3, YTHDF1, YTHDF2, COX-1, COX-2, and TBXAS1 were purchased from Abcam and used at a dilution of 1:1,000. The HRP-conjugated goat anti-rabbit IgG secondary antibody was purchased from Cell Signaling Technology (CST) and used at a dilution of 1:1,000.

### 2.10 Assessment of protein stability

Translational inhibition was achieved by the addition of 100 μg/mL CHX (MCE, New Jersey, USA) with an equal volume of PBS added to control groups and incubation for 10 h. Proteins were extracted for western blot analysis.

### 2.11 RNA immunoprecipitation assay

RNA immunoprecipitation (RIP) experiments were carried out using a RIP Kit (BersinBio, Guangzhou, China) on METTL3-overexpressing or METTL3-knockdown MEG-01 cells. Cells were treated with RNA lysis buffer, and incubated overnight at 4 °C in RIP buffer containing immobilized YTHDF1 antibody (Abcam) or negative control IgG before washing 3 times with lysis buffer. Samples were treated with proteinase K and immunoprecipitated RNA extracted with phenol-chloroform-isopentyl alcohol (25:24:1) mixture and *COX-1* mRNA was measured by qPCR.

### 2.12 Cell proliferation

Cell proliferation was measured with cell counting kit-8 (CCK-8; Dojindo, Kumamoto, Japan). MEG-01 cells were inoculated into a 96-well plate at a density of 1.2 × 10^4^ cells per well and transfected with METTL3-overexpressing or METTL3-knockdown lentiviruses for 48 or 72 h. CCK-8 solution (10 μL/well) was added for 2 h and absorbance was measured at 450 nm using a microplate reader (Synergy H1; Biotek).

### 2.13 Apoptosis

Apoptosis was assessed using the Annexin V-EGFP/PI kit (KeyGen Biotech), following the manufacturer’s instructions. Briefly, MEG-01 cells were seeded at a density of 1.2 × 10^4^ per well in a 96-well plate and transfected with METTL3-overexpressing or METTL3-knockdown lentiviruses for 24 or 48 h before harvesting by trypsinization and washing twice with cold PBS. Cells were incubated with Annexin V-EGFP and PI on ice for 5 min and analyzed by flow cytometry (LSR Fortessa™ X-20, BD). The percentages of viable (Annexin V−/PI−), early apoptotic (Annexin V+/PI−), late apoptotic (Annexin V+/PI+) and necrotic (Annexin V−/PI+) cells were determined.

### 2.14 Statistical analysis

Continuous variables were assessed for normality using the Shapiro-Wilk test. Normally distributed variables are presented as mean ± SD and were compared using the independent-samples t-test. Non-normally distributed variables are presented as median (Q1, Q3) and were compared using the Mann-Whitney U test. Categorical variables are presented as *n* (%) and were compared using Fisher’s exact test. Correlations were assessed using Spearman’s rank correlation analysis. For cell-based experiments, group comparisons were performed using independent-samples t-tests without adjustment for multiple comparisons, as specified in the figure legends. Given the exploratory nature and limited sample size of the clinical cohort, formal correction for multiple testing was not applied to the primary clinical correlation analyses; these *p*-values should therefore be interpreted cautiously and considered hypothesis-generating. Statistical analyses were performed using SPSS version 26.0 (SPSS Inc., Chicago, IL, USA). All tests were two-sided, and *p* < 0.05 was considered statistically significant. All cell-based experiments were performed in three independent biological replicates.

## 3 Results

### 3.1 Clinical data, m6A methylation and patient genomic modifications

A total of 34 inpatients routinely prescribed 100 mg/d aspirin were enrolled and no differences in age, sex, body mass index, comorbid conditions, biochemical parameters or pharmacological treatments were found between aspirin low-response and aspirin high-response groups (Table S2). For the patients in this study, the coadministered drugs included statins (38.23 % total, 57.14 % low-response, 25.00 % high-response, *p* = 0.080), ACE inhibitors (41.18 % total, 42.86 % low-response, 40.00 % high-response, *p* = 1.000), β-blockers (8.82 % total, 7.14 % low-response, 10.00 % high-response, *p* = 1.000), calcium channel blockers (23.53 % total, 28.57 % low-response, 20.00 % high-response, *p* = 0.689), and antidiabetic drugs (20.59 % total, 21.43 % low-response, 20.00 % high-response, *p* = 1.000). No statistically significant between-group differences were observed in coadministered medication use. However, given the modest sample size and the numerical imbalance in statin use, residual confounding cannot be excluded.

Total RNA was isolated from whole-blood samples, and overall m6A levels and the mRNA expression of seven m6A-related genes were assessed. m6A methylation was higher in the aspirin low-response than in the high-response group (*p* < 0.05, Figure 1A) and levels of *METTL3* (*p* < 0.01) and *YTHDF1* (*p* < 0.05) mRNA were also higher in the low-response than in the high-response group (Figure 1D). Increased serum levels of the AA metabolites PGH2 (*p* < 0.05) and TXB2 (*p* < 0.05) were also observed in the low-response group (Figure 1B and C).

**Figure 1:**
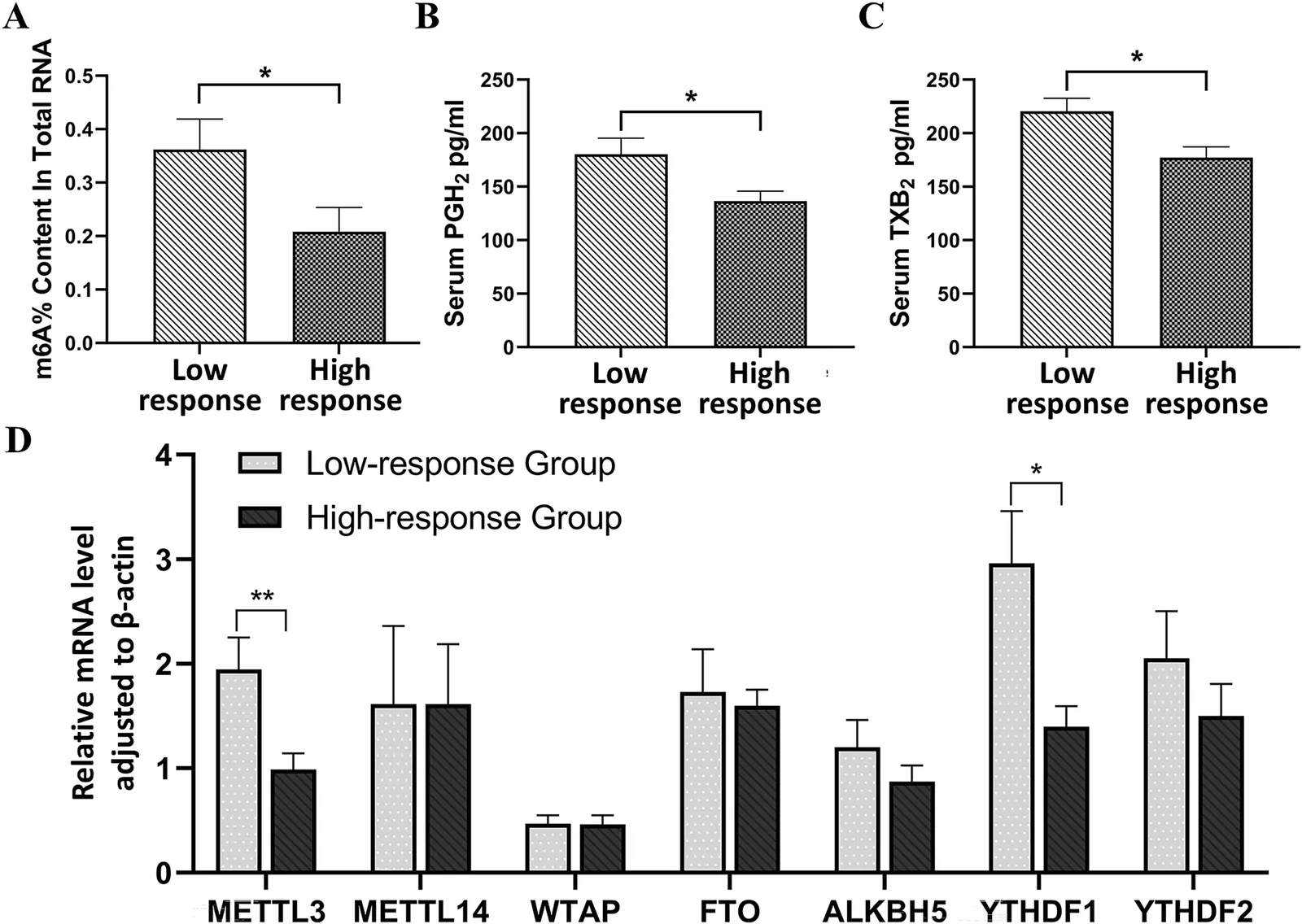
Whole blood levels of m6A RNA and expression of m6A-related genes in patients with low (*n* = 14) and high (*n* = 20) aspirin response. (A) Whole blood m6A RNA was detected by colorimetric assay; (B) serum PGH2 levels were measured by ELISA; (C) serum TXB2 levels were measured by ELISA; (D) the expression of 7 genes involved in m6A modification was evaluated by real-time quantitative polymerase chain reaction (qPCR). Data are expressed as mean ± SEM (*: *p* < 0.05; **: *p* < 0.01).

### 3.2 Correlation between m6A methylation-related genes and quantitative and qualitative patient indicators

The relationships between the expression of seven m6A methylation-related genes and quantitative and categorical clinical indicators were assessed by Spearman’s rank correlation analysis (Tables 1 and S3). *METTL3* and *YTHDF1* mRNA in blood were positively associated with PAR. Serum PGH2 was positively correlated with both *METTL3* and *YTHDF1* mRNA expression in peripheral blood, whereas serum TXB2 was positively correlated only with *METTL3* mRNA expression. Thus, the impact of *METTL3* and *YTHDF1* on aspirin response may be mediated by their interference with the conversion of AA to eicosanoids.

**Table 1:** Comparison of levels of mRNA expression of m6A-related genes and quantitative indicators among selected patients.

Variables	METTL3		METTL14		WTAP		FTO		ALKBH5		YTHDF1		YTHDF2	
	*r*_s_	*p*	*r*_s_	*p*	*r*_s_	*p*	*r*_s_	*p*	*r*_s_	*p*	*r*_s_	*p*	*r*_s_	*p*
Age	−0.041	0.816	0.273	0.118	0.187	0.290	0.180	0.308	0.102	0.566	−0.064	0.718	0.262	0.134
BMI	0.043	0.809	0.460	0.006*	0.298	0.086	0.218	0.216	−0.255	0.145	−0.276	0.127	−0.145	0.415
PAR	0.449	0.008*	0.069	0.699	0.033	0.854	−0.059	0.740	0.055	0.758	0.365	0.034*	0.072	0.687
White blood cell	−0.040	0.823	0.082	0.644	0.135	0.447	0.363	0.035*	−0.070	0.695	0.031	0.860	−0.056	0.754
Hemoglobin	−0.208	0.239	−0.003	0.987	0.191	0.280	0.126	0.478	0.331	0.056	0.098	0.581	0.224	0.202
Red blood cell	−0.167	0.345	−0.096	0.590	−0.022	0.904	0.119	0.503	0.258	0.140	0.238	0.176	0.282	0.106
Platelet	0.061	0.731	−0.012	0.948	0.000	0.998	0.196	0.266	−0.191	0.278	−0.179	0.311	−0.138	0.438
ALT	−0.083	0.640	0.133	0.452	0.085	0.635	−0.072	0.678	−0.054	0.760	−0.208	0.237	−0.121	0.496
AST	−0.024	0.894	0.051	0.773	0.103	0.564	−0.094	0.597	0.034	0.848	−0.262	0.134	−0.027	0.879
Serum creatinine	0.172	0.331	0.310	0.075	0.178	0.315	0.315	0.070	0.228	0.195	0.183	0.301	0.149	0.401
Serum uric acid	0.192	0.276	0.173	0.327	0.011	0.949	−0.009	0.959	0.010	0.956	0.013	0.943	0.050	0.778
FPG	−0.152	0.389	0.061	0.732	−0.173	0.328	0.073	0.681	0.040	0.821	−0.095	0.592	0.125	0.481
Triglyceride	−0.147	0.407	−0.051	0.774	0.132	0.456	0.081	0.650	−0.010	0.953	−0.022	0.902	−0.191	0.280
Total cholesterol	0.105	0.555	−0.051	0.776	−0.035	0.846	−0.383	0.025*	−0.316	0.096	−0.051	0.775	−0.130	0.462
HDL-C	0.101	0.568	−0.049	0.781	−0.241	0.169	−0.322	0.063	−0.115	0.518	−0.050	0.780	0.178	0.313
LDL-C	0.028	0.875	0.013	0.943	0.134	0.450	−0.256	0.144	−0.254	0.147	−0.127	0.474	−0.176	0.319
TXB_2_	0.385	0.025*	0.047	0.793	0.105	0.554	0.004	0.982	0.167	0.346	0.302	0.083	0.046	0.796
PGH_2_	0.393	0.021*	−0.187	0.288	0.012	0.947	−0.081	0.648	0.315	0.070	0.405	0.018*	0.214	0.223

BMI, body mass index; PAR, platelet aggregation rate; ALT, alanine aminotransferase; AST, aspartate transaminase; FPG, fasting plasma glucose; HDL-C, high-density lipoprotein cholesterol; LDL-C, low-density lipoprotein cholesterol; ACEIs, angiotensin-converting enzyme inhibitors; CCBs, calcium channel blockers; PGH_2_, prostaglandin H_2_; TXB_2_, thromboxane B_2_; *p < 0.05.

### 3.3 Validation of PMA-induced differentiation of MEG-01 cells

Before induction, MEG-01 cells displayed a rounded or irregular morphology, with relatively large nuclei, sparse cytoplasmic granules, and suspension growth as single cells or aggregates. Following PMA-induced differentiation, the cells exhibited megakaryocyte-like morphological changes, including increased cell size, elongated shapes, pseudopod-like projections, more abundant cytoplasmic granules, irregular lobulated nuclei, and partial adherence to the culture dish (Figure S2).

To optimize PMA-induced differentiation, MEG-01 cells were treated with PMA at concentrations of 0, 1, 5, 10, 15, 20, 40, 60, 80, 100, 200, and 400 ng/mL, and the surface expression of CD41 and CD61 was quantified by flow cytometry. The highest expression levels of CD41 and CD61 were observed after induction with 20 ng/mL PMA (Figures S3 and S4). MEG-01 cells were then induced with 20 ng/mL PMA for different durations (1, 2, 3, 4, 5, 6, 7, and 10 d). The highest surface expression of CD41 and CD61 was observed after 3 d of induction (Figures S5 and S6).

Under the optimal induction condition of 20 ng/mL PMA for 3 d, total RNA was extracted, and CD41 and CD61 mRNA expression was assessed by qPCR. Compared with untreated MEG-01 cells, CD41 and CD61 mRNA expression was significantly increased following PMA treatment (*p* < 0.001, Figure S7).

### 3.4 Effect of METTL3 on COX-1 expression

The validation of transfection efficiency of MEG-01 cells showed (Figure S8) that *METTL3* mRNA expression was up-regulated by more than 50 % in the OE group compared to the OE-NC group (*p* < 0.01), and *METTL3* mRNA expression was down-regulated by more than 30 % in the KD group compared to the KD-NC group (*p* < 0.05). Changes in mRNA and protein expression of key genes, linked to cardiovascular risk, were assessed by qPCR and western blotting following *METTL3* up- or downregulation. *TBXAS1* is required for synthesis of the platelet aggregator, thromboxane A2 (TXA2), *PTGIS* is required for synthesis of the anti-thrombotic and vasodilator, prostacyclin PGI2 and *PTGIR* are required for PGI2 activity. *METTL3* overexpression (OE) increased YTHDF1 mRNA (*p* < 0.01) and COX-1 protein (*p* < 0.05) compared with overexpressing negative controls (OE-NC) (Figure 2A–O). By contrast, *METTL3* downregulation (KD) decreased YTHDF1 and COX-1 mRNA and protein compared with downregulation-negative controls (KD-NC) (*p* < 0.05, Figure 2A–O). ELISA assays of cell culture supernatants were performed to evaluate the levels of PGH2 and TXB2. The PGH2 content of OE cells was higher than that of OE-NC cells after 120 h (*p* < 0.05, Figure 3A–C) and content of TXB2 was higher in OE cells at 48, 72 and 120 h (*p* < 0.05 or *p* < 0.01 Figure 3D–F). TXB2 was lower in KD cells than in KD-NC cells after 120 h (*p* < 0.05, Figure 3F). Upregulation of *METTL3* and consequent upregulation of *YTHDF1* and *COX-1* may have stimulated the synthesis of PGH2 and TXB2.

**Figure 2:**
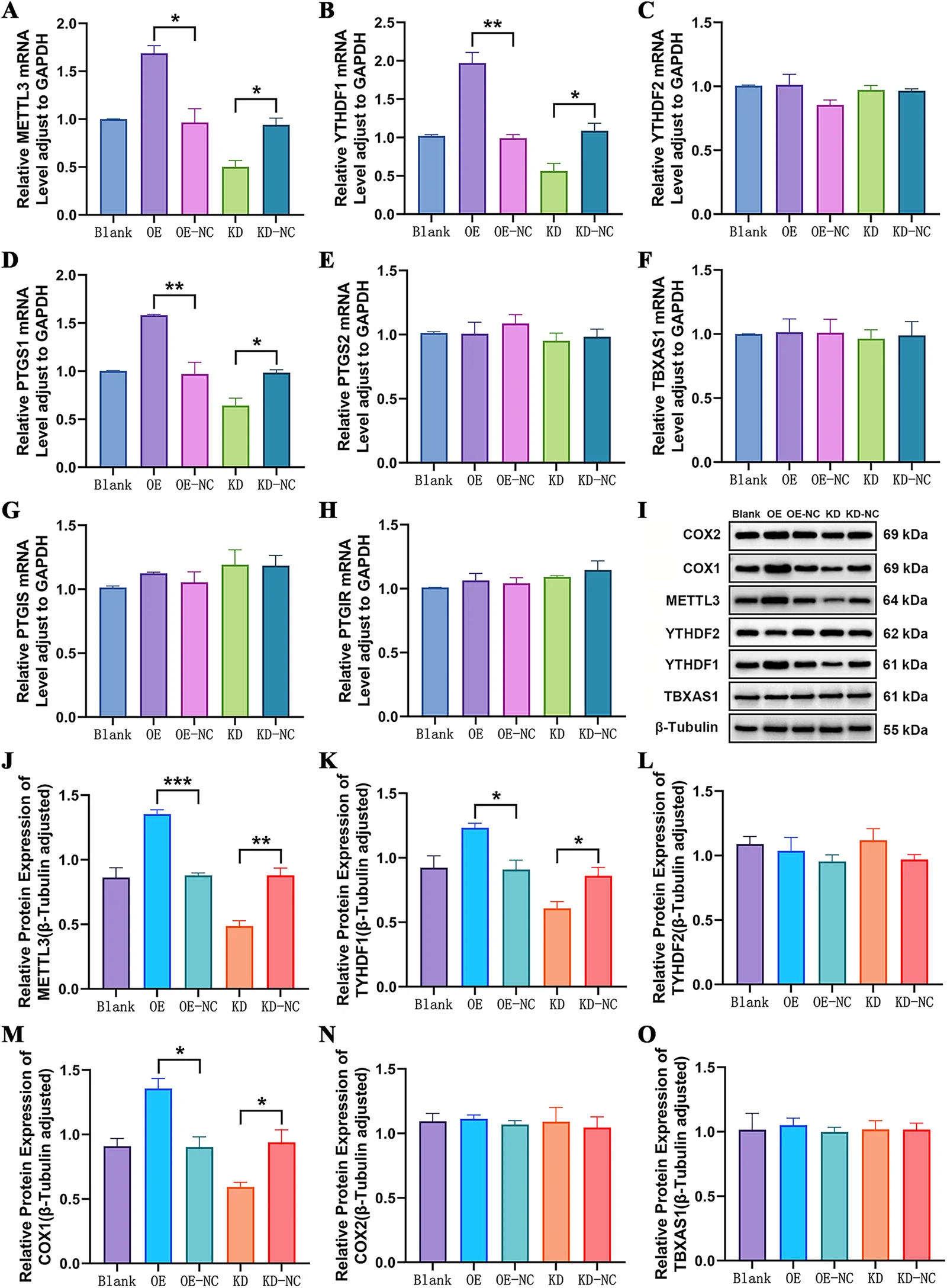
Effects of altered METTL3 expression on key genes involved in AA metabolism. (A–H) METTL3, YTHDF1, YTHDF2, COX-1 (PTGS1), COX-2 (PTGS2), TBXAS1, PTGIS and PTGIR mRNA expression measured by real-time qPCR; (I–O) METTL3, YTHDF1, YTHDF2, COX-1, COX-2 and TBXAS1 protein expression measured by western blotting. Data are expressed as mean ± SEM (*: *p* < 0.05; **: *p* < 0.01; ***: *p* < 0.001; ns: *p* > 0.05). All experiments were repeated three times. Comparisons between the indicated groups were performed using independent-samples t-tests.

**Figure 3:**
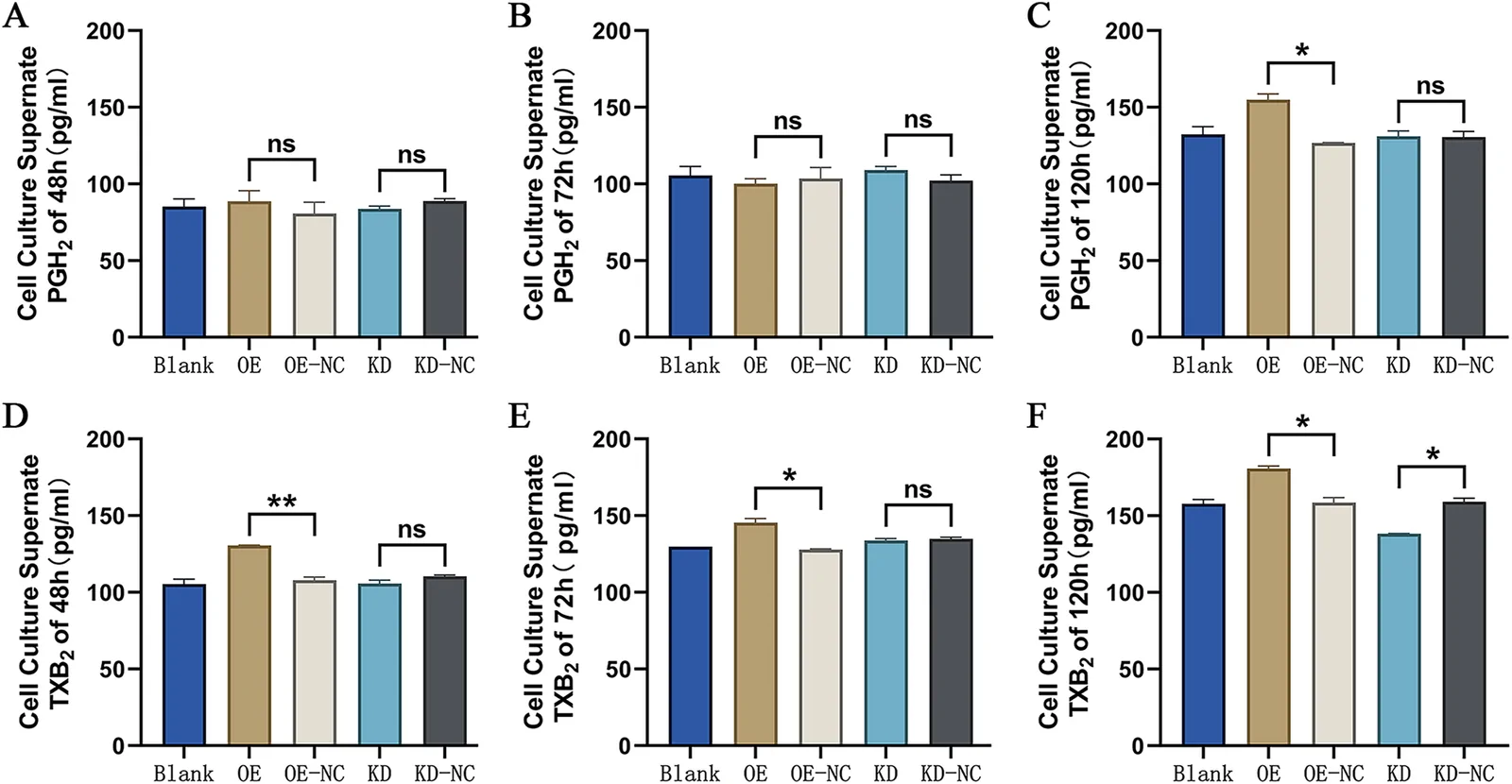
Extracellular PGH2 and TXB2 levels in cell-culture supernatants over time. (A–C) PGH2 levels in cell-culture supernatants measured by ELISA at 48, 72, and 120 h; (D–F) TXB2 levels in cell-culture supernatants measured by ELISA at 48, 72, and 120 h. Data are presented as mean ± SEM. Comparisons between the indicated groups were performed using independent-samples t-tests. *: *p* < 0.05; **: *p* < 0.01; ns: not significant.

### 3.5 METTL3 promoted translation of COX-1 mRNA via YTHDF1

*METTL3* upregulation did not increase levels of *COX-1* protein (Figure 4A) after treatment with the inhibitor of translation, cycloheximide (CHX), suggesting that the observed effect of *METTL3* on *COX-1* expression may be due to translational regulation. *YTHDF1* recognizes m6A modifications of target genes and binds to the translational initiation complex to enhance translation. *METTL3* upregulation increased *COX-1* expression (Figure 4B) and *METTL3* downregulation decreased expression (Figure 4A). However, *METTL3* upregulation in the presence of *YTHDF1* downregulation failed to produce an increase in *COX-1* expression (Figure 4B). Thus, *YTHDF1* may be involved in translational regulation of COX-1 mRNA. RNA immunoprecipitation (IP) experiments showed that the addition of anti-*YTHDF1* antibody precipitated a larger amount of COX-1 mRNA than the addition of the control non-specific IgG (*p* < 0.01 or *p* < 0.001, Figure 4C), indicating binding of *YTHDF1* to COX-1 mRNA. *METTL3* upregulation increased the content of COX-1 mRNA in the OE-IP subgroup compared with the OE-NC-IP subgroup (*p* < 0.01, Figure 4C). *METTL3* downregulation had the opposing effect with lower COX-1 mRNA content in the KD-IP subgroup compared with the KD-NC-IP subgroup (*p* < 0.01, Figure 4C). Thus, *METTL3* upregulation promoted *YTHDF1* binding to COX-1 mRNA due to the increased degree of m6A modification. We conclude that this sequence of events is responsible for the enhanced translation of COX-1 mRNA and increase in *COX-1* protein.

**Figure 4:**
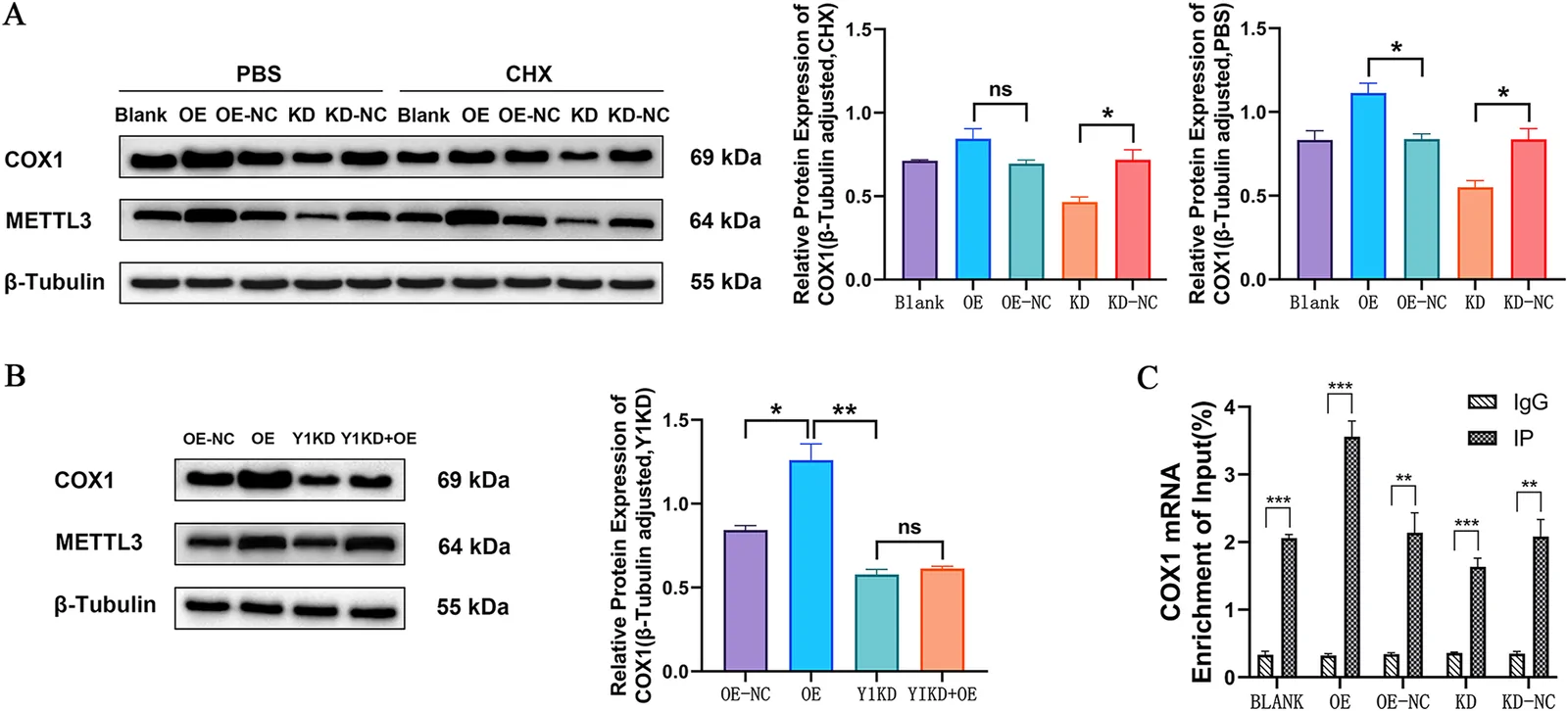
METTL3 promoted the translation of COX-1 mRNA through YTHDF1-dependent mechanism. (A) Western blots of COX-1 protein after up- and downregulation of METTL3 in the presence or absence of CHX; (B) western blots of COX-1 protein following METTL3 upregulation with YTHDF1 downregulation; (C) RNA IP experiments with anti-YTHDF1 antibodies. Data are expressed as mean ± SEM (*: *p* < 0.05; **: *p* < 0.01; ***: *p* < 0.001; ns: *p* > 0.05). All experiments were repeated three times. Comparisons between the indicated groups were performed using independent-samples t-tests.

### 3.6 Impact of METTL3 on megakaryocyte function

Megakaryocyte proliferation was increased 48 h after transfection in OE cells compared with OE-NC cells (Figure 5A). However, no differences were found between KD and KD-NC cells (Figure 5A). Proliferation rates were also higher in OE than OE-NC cells at 72 h (Figure 5B) but were reduced in KD compared with KD-NC cells (Figure 5B). It is concluded that METTL3 upregulation promoted megakaryocyte proliferation and downregulation inhibited proliferation. In the flow cytometry plots, KD cells showed a higher proportion of apoptotic cells than KD-NC cells, while OE and OE-NC cells showed similar proportions (Figure 5C). These observations suggest that METTL3 downregulation may be associated with increased megakaryocyte apoptosis.

**Figure 5:**
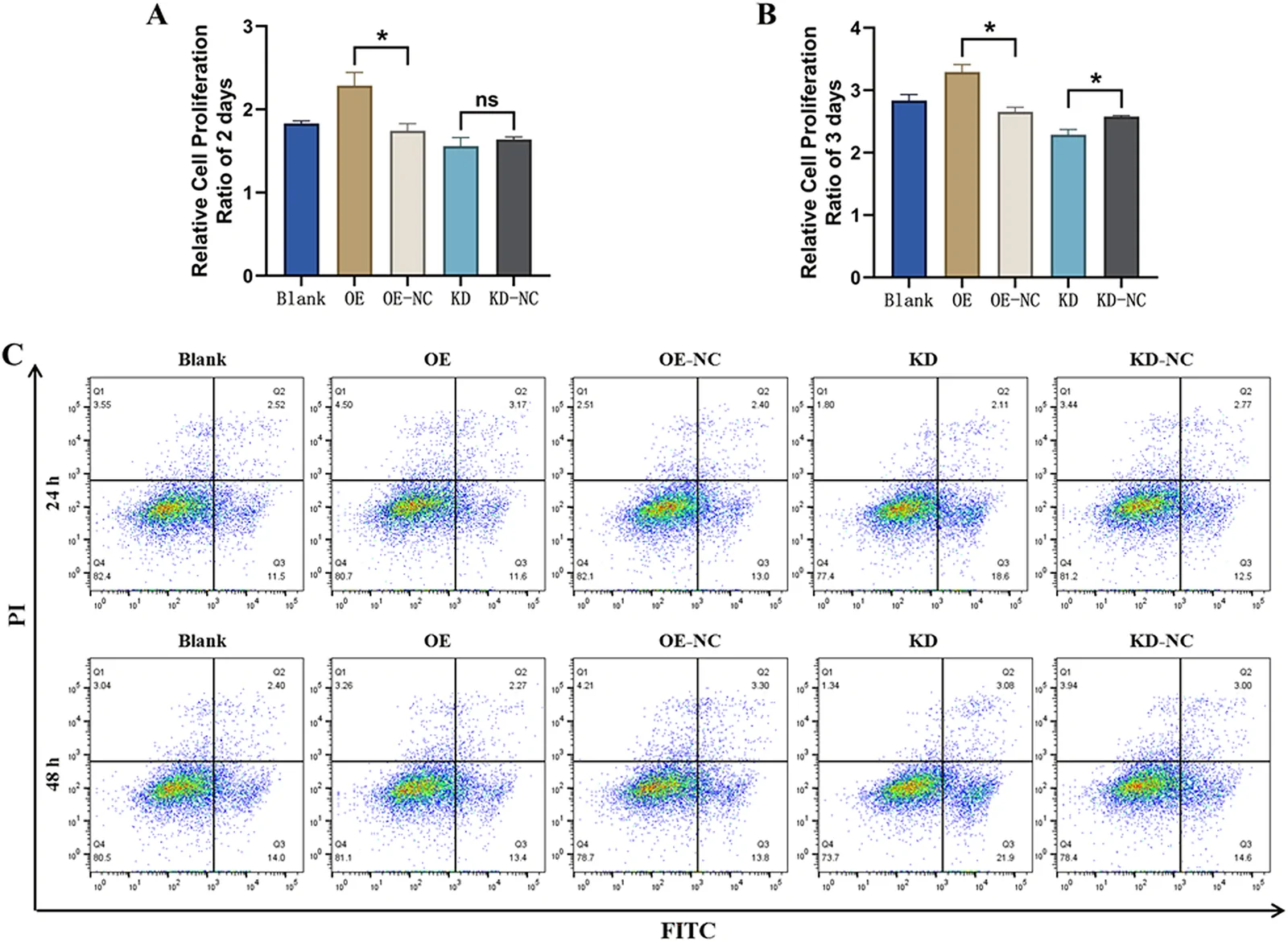
Effects of METTL3 on MEG-01 cell proliferation and apoptosis. (A) Proliferation of MEG-01 cells following METTL3-overexpressing or METTL3-knockdown lentiviral transfection for 48 h, evaluated using the CCK-8 assay; (B) proliferation of MEG-01 cells following METTL3-overexpressing or METTL3-knockdown lentiviral transfection for 72 h evaluated using the CCK-8 assay; (C) apoptotic status 24 and 48 h after transfection with METTL3-overexpressing or METTL3-knockdown lentivirus determined by Annexin V-EGFP/PI staining and flow cytometry. Data are expressed as mean ± SEM (*: *p* < 0.05; ns: *p* > 0.05). All experiments were repeated three times. Comparisons between the indicated groups in panels (A) and (B) were performed using independent-samples t-tests.

## 4 Discussion

The present study aimed to investigate whether m6A-related post-transcriptional regulation is associated with reduced antiplatelet responsiveness to aspirin in older adults. We found that patients with low aspirin response showed higher whole-blood m6A levels, increased METTL3 and YTHDF1 expression, and elevated PGH2 and TXB2 levels. In MEG-01 cells, METTL3 overexpression increased YTHDF1 and COX-1 expression, promoted PGH2/TXB2 production, and affected megakaryocytic proliferation and apoptosis, whereas METTL3 knockdown showed the opposite tendency. Mechanistically, CHX and RIP assays supported a model in which METTL3 enhances YTHDF1-dependent binding to COX-1 mRNA and promotes COX-1 translation. These findings suggest that the METTL3/YTHDF1/COX-1 axis may contribute to aspirin low responsiveness by modulating arachidonic acid metabolism and megakaryocyte-related functions. Previous studies have shown that immune-inflammatory and oxidative-stress pathways can alter the functional state of peripheral immune cells and disease susceptibility, including pathways involving Toll-like receptors [30], NF-κB-related signaling [31], IL-6/IL-23/Th17 responses [32], and dysregulated antioxidant networks. Although these studies were conducted in different disease contexts and do not directly establish a link with aspirin responsiveness, they support the broader concept that systemic inflammatory and redox states may influence cellular responses. In cardiovascular disease, such immune-inflammatory and oxidative milieus may interact with platelet activation, arachidonic acid metabolism, and antiplatelet responsiveness. Therefore, the METTL3/YTHDF1/COX-1 axis identified in the present study should be interpreted within this broader biological context and further validated in larger clinical and mechanistic studies.

The present study correlated levels of m6A modifications in whole blood RNA with patient aspirin response. METTL3 and YTHDF1 were hypothesized to affect this response by influencing the pathways of conversion of AA into eicosanoid hormones. METTL3 upregulation increased YTHDF1 expression *in vitro*, enhancing the binding of YTHDF1 to m6A-modified COX-1 mRNA due to the increased modification. The result was increased translation of COX-1 mRNA and increased levels of COX-1 protein which affected the aspirin response. The possibility that METTL3 affected the aspirin response by influencing megakaryocytic function and platelet production was also investigated (Figure 6). To the best of our knowledge, the current work represents the first association of m6A methylation, METTL3 with the aspirin response and AA metabolism.

**Figure 6:**
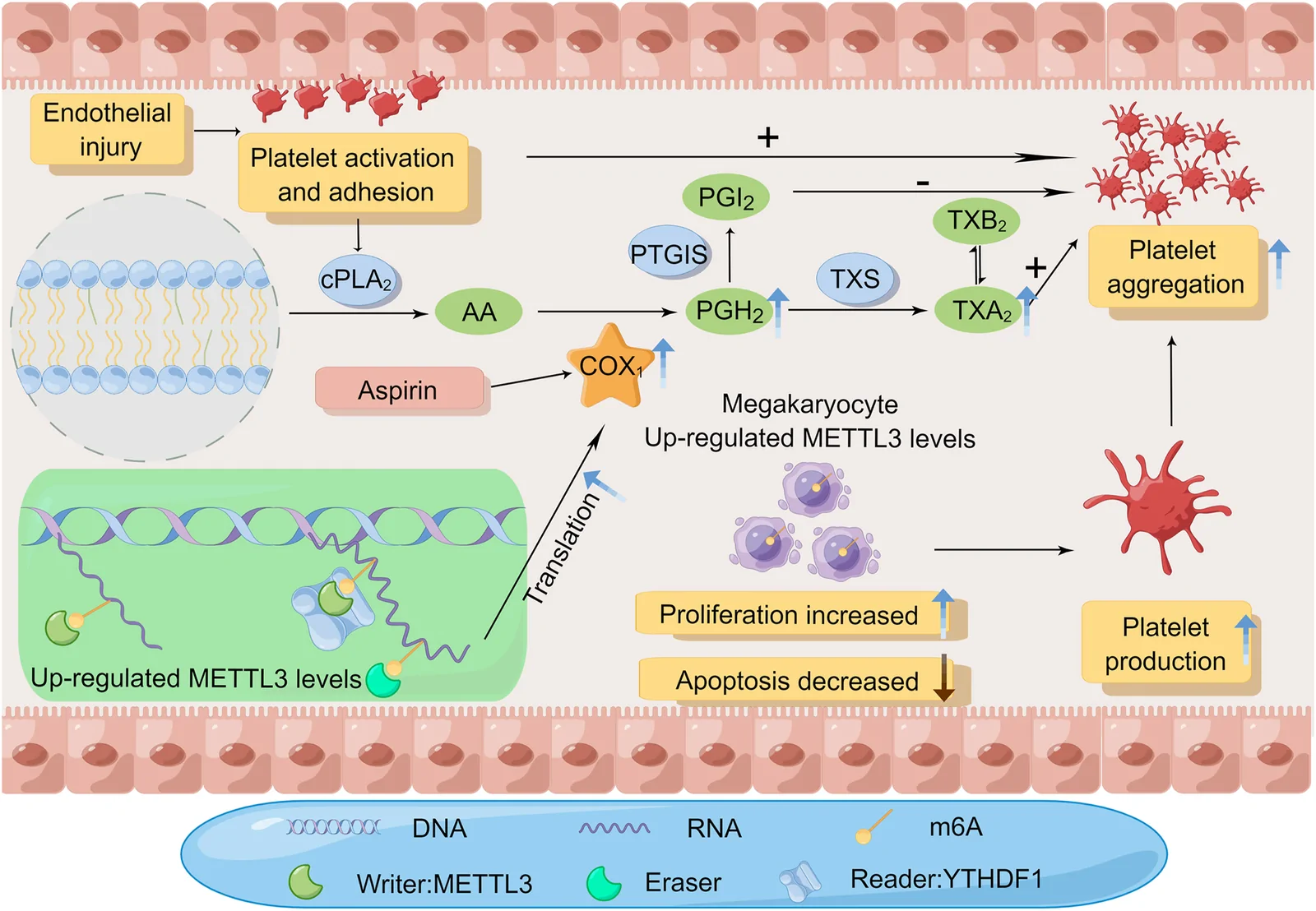
Schematic diagram illustrating the mechanism of the METTL3-mediated YTHDF1-COX-1 axis in reducing the antiplatelet efficiency of aspirin in older adults.

In this study, METTL3 upregulation enhances COX-1 translation efficiency and baseline expression, but whether it alters COX-1’s spatial conformation or its susceptibility to aspirin-mediated acetylation requires structural validation. While serum TXB_2_ is widely accepted as a surrogate for platelet-derived TXA_2_, we cannot entirely exclude extra-platelet contributors. For example, COX-2 derived from inflammatory monocytes or vascular endothelial cells, can actively synthesize residual TXA_2_, thereby stimulating pathways of residual platelet activation that escape standard aspirin suppression. Given that METTL3 promotes megakaryocyte proliferation, it raises the question of whether aspirin low-responsiveness stems from an increased density of COX-1 molecules within each individual platelet, or rather from an accelerated platelet turnover rate. Future investigations utilizing mass spectrometry-based turnover assays and single-platelet proteomics are highly warranted to fully disentangle these intra-platelet and systemic dynamics.

Various means of testing platelet function to evaluate aspirin response are available but a unified standard for the diagnosis of low-response to aspirin has yet to be established [33], 34]. LTA is considered the gold standard for measuring AA-induced platelet aggregation and we have previously shown patients with low-response to aspirin diagnosed by LTA-detected PAR to be more prone to cardiovascular events [33], [35], [36], [37]. LTA was used in the current study to assess PAR and aspirin response and this method is widely recognized as an approach to treatment optimization for patients with low-response to aspirin.

Platelets are anucleate cell fragments produced by megakaryocytes and are difficult to propagate *in vitro*, resulting in few studies on long-term platelet culture [38]. The most commonly utilized cell line for studies of platelet function is the human megakaryocytic leukemia cell line, MEG-01 [39], 40]. PMA was used in the current work to stimulate the differentiation of MEG-01 cells into mature megakaryocytes and CD41 and CD61 were detected on both MEG-01 cells and platelets by flow cytometry. The optimal conditions for PMA-induced MEG-01 differentiation were established and included induction with 20 ng/mL PMA for 3 d. We are confident that MEG-01 cells are suitable substitutes for platelets and allowed us to construct a cell model of differential METTL3 expression. As COX-2 is a major contributor to eicosanoid biosynthesis in endothelial and epithelial cells, future studies should be conducted to investigate whether METTL3 and YTHDF1 modulate COX-1 and COX-2 expression in these cells so as to influence aspirin’s effects, thereby extending the conclusions of this study.

m6A modifications have been associated with cardiovascular disease and the consequent transcriptional effects may influence atherosclerosis and angiogenesis [25], 27]. However, the influence of m6A modification on AA metabolism, platelet formation and aspirin response has been little studied. The current study established the connection between m6A modification and aspirin response. METTL3 affected the aspirin response by facilitating the YTHDF1-dependent translation of COX-1 in the pathway of conversion of AA to eicosanoids. Platelet aggregation is strongly affected by platelet formation since these cell fragments are unable to replicate [38]. METTL3 influenced MEG-01 proliferation and apoptosis and the resulting effect on platelet generation and function may affect the aspirin response.

We acknowledge some limitations to the current work. First, LTA was the only technique used to assess PAR in a small sample size for the evaluation of aspirin response. Additionally, although co-medications were overall balanced, statin use showed a non-significant numerical disparity between groups (*p* = 0.080), which might introduce subtle residual confounding given the known pleiotropic effects of statins on megakaryocytes and endothelial function. Second, because formal multiple-testing correction was not applied to the primary clinical correlation analyses, the possibility of false-positive associations cannot be excluded. The observed correlations should therefore be interpreted as exploratory and require validation in larger independent cohorts. Third, the potential effects and mechanisms of METTL3 on aspirin response were investigated from the perspective of AA metabolism and megakaryocyte function but the mechanisms by which altered METTL3 expression affects YTHDF1 expression, including any potential positive-feedback regulation, were not explored. Furthermore, the present study mainly focused on post-transcriptional and translational regulation mediated by the METTL3/YTHDF1/COX-1 axis. We did not systematically investigate upstream transcriptional signaling pathways or transcription factor-mediated regulation. Therefore, future studies using transcriptome-wide profiling, m6A-seq, and transcriptional regulatory analyses are needed to determine whether broader transcriptional signaling networks interact with METTL3/YTHDF1-mediated COX-1 regulation in aspirin low responsiveness. Fourth, the effect of METTL3 on megakaryocyte proliferation and apoptosis was assessed but not the impact on megakaryocyte-generated platelets or platelet activity. Fifth, the effects of METTL3 on the aspirin response *in vitro* were examined but animal experiments are required to explore the actions *in vivo*. Sixth, the measurement of TXB2 and PGH2 by enzyme-linked immunosorbent assay (ELISA) without the standard 37 °C, 60 min whole blood incubation may produce artifactual TXB2 levels and unreliable PGH2 data due to its instability. Future studies should apply the gold standard TXB2 assay to strengthen our conclusions and explore TXB2 levels as a potential classification index for aspirin response to validate the findings. Our findings regarding the METTL3-YTHDF1-COX-1 axis may offer a valuable conceptual framework for future biomarker development and therapeutic stratification. In clinical practice, evaluating peripheral blood m6A modification profiles or specific METTL3 expression levels might eventually serve as a novel non-invasive biomarker panel to screen for individuals predisposed to aspirin low-responsiveness before major ischemic events occur. Furthermore, this pathway could inform future therapeutic stratification strategies; elderly patients identified with high METTL3-driven COX-1 translation might benefit more from tailored antiplatelet regimens, such as a combination therapy with adenosine diphosphate (ADP) receptor inhibitors or alternative lipid-pathway modulators, rather than increasing the dose of aspirin which elevates bleeding risks. However, these translational insights remain speculative at this preliminary stage, and robust, multi-center prospective studies are mandatory to verify the clinical utility of this axis. Moreover, the small clinical sample size may increase the risk of type I error and limits the stability of correlation estimates. Although MEG-01 cells provide a useful megakaryocytic model, platelet aggregation or thromboxane-generation assays using platelets derived from METTL3-modified megakaryocytes were not performed. Potential off-target effects of lentiviral overexpression or knockdown cannot be completely excluded, although negative-control lentiviral vectors were used.

## 5 Conclusions

In summary, our study demonstrates that the METTL3-associated YTHDF1-COX-1 axis plays a critical role in modulating arachidonic acid metabolism and megakaryocyte function, thereby contributing to reduced antiplatelet responsiveness to aspirin in older adults. By bridging m6A epitranscriptomic dynamics with downstream prothrombotic lipid synthesis, these findings provide a novel mechanistic framework to explain the inter-individual heterogeneity of aspirin efficacy. Clinically, this newly identified epigenetic axis is a potential biomarker for predicting aspirin low-responsiveness and may offer a novel therapeutic target to optimize precision antiplatelet strategies in aging populations.

## Supplementary Material

Supplementary Material
