# Supplementary material for: METTL3-associated YTHDF1-COX-1 axis is linked to reduced antiplatelet response to aspirin in older adults
Source: Open Life Sci. 2026 Nov 6;21(1):20251375. doi: 10.1515/biol-2025-1375 (PMC13738320; doi:10.1515/biol-2025-1375)
Supplement: Supplementary file 1 [file j_biol-2025-1375_suppl_001.docx]

**Supplementary materials**

**Figure S1:** Antiplatelet activity of aspirin. cPLA_2_: cytosolic phospholipase A_2_; AA: arachidonic acid; COX_1_: cyclooxygenase 1; PGH_2_: prostaglandin H_2_; PGI_2_: prostaglandin I_2_; TXA_2_: thromboxane A_2_; TXB_2_: thromboxane B_2_ (a stable metabolite of TXA_2_); PTGIS: prostaglandin I_2_ synthase; TXS: thromboxane synthase; ADP: adenosine diphosphate.

**Figure S2:** Microscopic morphology of MEG-01 cells before and after PMA induction. A. Microscopic morphology of MEG-01 cells; B. Microscopic morphology of MEG-01 cells treated with 20 ng/mL PMA for three days; C. Microscopic morphology of MEG-01 cells after Riehl's staining; D. Microscopic morphology of MEG-01 cells after Riehl's staining of MEG-01 cells treated with 20 ng/mL PMA for three days. The images were observed and recorded under an ordinary light microscope at 100×, and the length of the scale in the figure represents 100 μm.

**Figure S3:** CD41 expression on the surface of MEG-01 cells after treatment with different concentrations of PMA.

**Figure S4:** CD61 expression on the surface of MEG-01 cells after treatment with different concentrations of PMA.

**Figure S5:** CD41 expression on the surface of MEG-01 cells after 20 ng/mL PMA treatment for different times.

**Figure S6:** CD61 expression on the surface of MEG-01 cells after 20 ng/mL PMA treatment for different times.

**Figure S7:** Comparison of CD41 and CD61 mRNA expression levels before and after induction of MEG-01 cells. Note: ***: *p* < 0.001, the difference is statistically significant. Comparisons between the untreated and PMA-treated groups were performed using independent-samples t-tests.

**Figure S8:** Intergroup comparison of METTL3 mRNA in each group of cells after transfection. Note: **: *p* < 0.01, the difference is statistically significant; *: p < 0.05, the difference is statistically significant. Comparisons between the indicated groups were performed using independent-samples t-tests.

**Table S1:** PCR primer sequences

| **Primer** | **Sequence (5′ to 3′)** | **Product length (bp)** |
| --- | --- | --- |
| β-actin Forward | CACGGCTGCTTCCAGCTC | 134 |
| β-actin Reverse | CACAGGACTCCATGCCCAG |  |
| GAPDH Forward | TGCACCACCAACTGCTTAGC | 87 |
| GAPDH Reverse | GGCATGGACTGTGGTCATGAG |  |
| CD41 Forward | GATGAGACCCGAAATGTAGGC | 137 |
| CD41 Reverse | GTCTTTTCTAGGACGTTCCAGTG |  |
| CD61 Forward | CATGAAGGATGATCTGTGGAGC | 85 |
| CD61 Reverse | AATCCGCAGGTTACTGGTGAG |  |
| METTL3 Forward | AGATGGGGTAGAAAGCCTCCT | 111 |
| METTL3 Reverse | TGGTCAGCATAGGTTACAAGAGT |  |
| METTL14 Forward | GAACACAGAGCTTAAATCCCCA | 99 |
| METTL14 Reverse | TGTCAGCTAAACCTACATCCCTG |  |
| WTAP Forward | CTTCCCAAGAAGGTTCGATTGA | 158 |
| WTAP Reverse | TCAGACTCTCTTAGGCCAGTTAC |  |
| FTO Forward | ACTTGGCTCCCTTATCTGACC | 145 |
| FTO Reverse | TGTGCAGTGTGAGAAAGGCTT |  |
| ALKBH5 Forward | ACGGATCCTGGAGATGGACA | 162 |
| ALKBH5 Reverse | ATCTTCACCTTTCGGGCAGG |  |
| YTHDF1 Forward | GGGGACAAGTGGGTCTCAAG | 126 |
| YTHDF1 Reverse | AGGGTGTCGCTGTGAAAGC |  |
| YTHDF2 Forward | CCTTAGGTGGAGCCATGATTG | 118 |
| YTHDF2 Reverse | TCTGTGCTACCCAACTTCAGT |  |
| PTGS1 Forward | GCACCAACCTCATGTTTGCC | 195 |
| PTGS1 Reverse | CCAGCACCTGGTACTTGAGTT |  |
| PTGS2 Forward | GTTCCACCCGCAGTACAGAA | 106 |
| PTGS2 Reverse | AGGGCTTCAGCATAAAGCGT |  |
| TBXAS1 Forward | CAACACCAGCCCAGCCCTAT | 55 |
| TBXAS1 Reverse | GGCCCACAATCTCATCCACA |  |
| PTGIS Forward | CTTCCACCAATTCCCACTGCT | 159 |
| PTGIS Reverse | AGCAAAGTGCCAGCAACCAC |  |
| PTGIR Forward | CTCTCACGATCCGCTGCTTC | 99 |
| PTGIR Reverse | GGATGGGGTTGAAGGCGTAG |  |

**Table S2:** Clinical and laboratory parameters of participants

| **Variables** | **Total (n = 34)** | | **Low-response (n = 14)** | | **High-response (n = 20)** | | ***p*-Value** | |
| --- | --- | --- | --- | --- | --- | --- | --- | --- |
| Age (y) | | 66 (62-70) | | 64.50 (61.00-66.25) | | 66.50 (63.75-71.00) | | 0.112 |
| Male [n (%)] | | 14 [41.18 %] | | 6 [42.86 %] | | 8 [40.00 %] | | 1.000 |
| BMI (Kg/m^2^) | | 25.59 (23.68-26.94) | | 25.49 (23.60-28.52) | | 25.59 (23.53-26.86) | | 0.691 |
| PAR (%) | | 4.81 (2.61-16.33) | | 17.54 (15.01-22.91) | | 3.41 (1.73-4.52) | | ＜0.001^***^ |
| White blood cell (10^9^/L) | | 5.96 ± 1.23 | | 6.03 ± 1.21 | | 5.91 ± 1.27 | | 0.779 |
| Hemoglobin (g/L) | | 143.35 ± 13.31 | | 142.93 ± 15.59 | | 143.65 ± 11.87 | | 0.879 |
| Red blood cell (10^12^/L) | | 4.74 ± 0.44 | | 4.71 ± 0.53 | | 4.76 ± 0.38 | | 0.725 |
| Platelet (10^9^/L) | | 241.91 ± 54.57 | | 230.50 ± 66.50 | | 249.90 ± 44.53 | | 0.315 |
| ALT (IU/L) | | 20.60 (16.50-26.50) | | 22.60 (17.98-28.28) | | 20.50 (15.00-25.00) | | 0.377 |
| AST (IU/L) | | 22.20 ± 4.56 | | 21.71 ± 4.36 | | 22.55 ± 4.78 | | 0.598 |
| Serum creatinine (µmol/L) | | 65.88 ± 18.48 | | 69.57 ± 19.34 | | 63.3 ± 17.90 | | 0.338 |
| Serum uric acid (µmol/L) | | 315.12 ± 68.92 | | 318.29 ± 88.92 | | 312.90 ± 53.19 | | 0.827 |
| FPG (mmol/L) | | 5.40 (4.88-6.05) | | 5.14 (4.63-5.84) | | 5.45 (4.95-6.15) | | 0.259 |
| Triglyceride (mmol/L) | | 1.61 (0.95-2.11) | | 1.62 (1.11-2.11) | | 1.49 (0.92-2.17) | | 0.522 |
| Total cholesterol (mmol/L) | | 5.23 (4.52-6.22) | | 5.02 (4.36-5.96) | | 5.62 (4.53-6.23) | | 0.436 |
| HDL-C (mmol/L) | | 1.45 (1.23-1.80) | | 1.34 (1.15-1.91) | | 1.51 (1.32-1.74) | | 0.691 |
| LDL-C (mmol/L) | | 3.20 ± 0.97 | | 3.05 ± 1.07 | | 3.47 ± 0.80 | | 0.453 |
| Statins [n (%)] | | 13 [38.23 %] | | 8 [57.14 %] | | 5 [25.00 %] | | 0.080 |
| ACEIs [n (%)] | | 14 [41.18 %] | | 6 [42.86 %] | | 8 [40.00 %] | | 1.000 |
| β-blockers [n (%)] | | 3 [8.82 %] | | 1 [7.14 %] | | 2 [10.00 %] | | 1.000 |
| CCBs [n (%)] | | 8 [23.53 %] | | 4 [28.57 %] | | 4 [20.00 %] | | 0.689 |
| Antidiabetics [n (%)] | | 7 [20.59 %] | | 3 [21.43 %] | | 4 [20.00 %] | | 1.000 |
| Hypertension [n (%)] | | 25 [73.53 %] | | 11 [78.57 %] | | 14 [70.00 %] | | 0.704 |
| Diabetes [n (%)] | | 7 [20.59 %] | | 3 [21.43 %] | | 4 [20.00 %] | | 1.000 |
| Dyslipidemia [n (%)] | | 23 [67.65 %] | | 9 [64.29 %] | | 14 [70.00 %] | | 1.000 |

Note: Continuous variables are presented as mean ± SD or median (Q1, Q3), as appropriate; categorical variables are presented as n (%). BMI: body mass index; PAR: platelet aggregation rate; ALT: alanine aminotransferase; AST: aspartate transaminase; FPG: fasting plasma glucose; HDL-C: high-density lipoprotein cholesterol; LDL-C: low-density lipoprotein cholesterol; ACEIs: angiotensin-converting enzyme inhibitors; CCBs: calcium channel blockers. Hypertension: systolic blood pressure ≥140 mmHg and/or diastolic blood pressure ≥90 mmHg, or current use of antihypertensive medication; Diabetes: FPG ≥7.0 mmol/L or 2-h postprandial blood glucose ≥11.1 mmol/L; Dyslipidemia: total cholesterol ≥5.2 mmol/L, low-density lipoprotein cholesterol ≥3.4 mmol/L, triglycerides ≥1.7 mmol/L, or current use of lipid-lowering medication. ***p < 0.001.

**Table S3:** Comparison of the levels of mRNA expression of m6A-related genes and qualitative indicators among selected patients

| Variables | n | METTL3 | | METTL14 | | WTAP | | FTO | | ALKBH5 | | YTHDF1 | | YTHDF2 | |
| --- | --- | --- | --- | --- | --- | --- | --- | --- | --- | --- | --- | --- | --- | --- | --- |
|  |  | Expression | *p* | Expression | *p* | Expression | *p* | Expression | *p* | Expression | *p* | Expression | *p* | Expression | *p* |
| Sex |  |  | 0.743 |  | 0.592 |  | 0.691 |  | 0.522 |  | 0.743 |  | 0.691 |  | 0.192 |
| Men | 14 | 1.24 ± 0.84 |  | 0.58  (0.16, 1.65) |  | 0.47 ± 0.30 |  | 1.50  (0.94, 2.29) |  | 0.83  (0.28, 1.04) |  | 1.51  (0.73, 2.63) |  | 0.95  (0.28, 2.15) |  |
| Women | 20 | 1.25  (0.63, 2.02) |  | 0.32  (0.13, 2.52) |  | 0.34  (0.15，0.78) |  | 1.27  (0.89, 1.92) |  | 0.78  (0.39, 1.99) |  | 1.45  (0.94, 2.97) |  | 1.67  (0.72, 2.80) |  |
| Statins |  |  | 0.506 |  | 0.326 |  | 0.039* |  | 0.552 |  | 0.181 |  | 0.462 |  | 0.292 |
| Yes | 13 | 1.48 ± 1.01 |  | 0.76  (0.19, 1.65) |  | 0.63 ± 0.40 |  | 1.58  (0.83, 2.97) |  | 1.15 ± 0.72 |  | 1.64 ± 1.01 |  | 2.03 ± 1.60 |  |
| No | 21 | 0.89  (0.60, 1.93) |  | 0.31  (0.13, 3.10) |  | 0.30  (0.15, 0.52) |  | 1.29  (0.93, 1.79) |  | 0.68  (0.27, 0.97) |  | 1.69  (0.87, 3.76) |  | 0.94  (0.43, 2.57) |  |
| ACEIs |  |  | 0.083 |  | 0.904 |  | 0.666 |  | 0.5 |  | 0.904 |  | 0.377 |  | 0.823 |
| Yes | 14 | 0.99 ± 0.67 |  | 0.72  (0.13, 1.48) |  | 0.28  (0.15, 0.79) |  | 1.65 ± 0.83 |  | 0.93 ± 0.63 |  | 1.26  (0.84, 2.51) |  | 1.68 ± 1.29 |  |
| No | 20 | 1.66 ± 1.13 |  | 0.32  (0.14, 2.52) |  | 0.39  (0.18, 0.73) |  | 1.27  (0.90, 1.81) |  | 0.73(0.31 ,1.81) |  | 2.19 ± 1.55 |  | 1.15  (0.49, 2.75) |  |
| β-blockers |  |  | 0.645 |  | 0.262 |  | 0.289 |  | 0.136 |  | 0.645 |  | 0.819 |  | 0.524 |
| Yes | 3 | 1.07 ± 1.11 |  | 1.74 ± 1.78 |  | 0.23 ± 0.11 |  | 2.10 ± 0.48 |  | 1.16 ± 1.02 |  | 2.61 ± 3.30 |  | 2.79 ± 2.49 |  |
| No | 31 | 1.01  (0.67, 2.00) |  | 0.33  (0.14, 2.24) |  | 0.39  (0.18, 0.79) |  | 1.25  (0.92, 1.84) |  | 0.79  (0.37, 1.53) |  | 1.51  (0.91, 2.68) |  | 1.12  (0.54, 2.62) |  |
| CCBs |  |  | 0.347 |  | 0.288 |  | 0.647 |  | 0.27 |  | 0.39 |  | 0.618 |  | 0.084 |
| Yes | 8 | 1.54 ± 0.81 |  | 0.86  (0.19, 3.63) |  | 0.37±0.25 |  | 1.66  (1.14, 2.98) |  | 1.34 ± 1.05 |  | 2.03 ± 1.27 |  | 2.88 ± 2.16 |  |
| No | 26 | 0.93  (0.61, 2.03) |  | 0.34  (0.14, 1.35) |  | 0.38  (0.15,  0.80) |  | 1.19  (0.91, 2.01) |  | 0.79  (0.34, 1.04) |  | 1.41  (0.85,  2.89) |  | 1.06  (0.43, 2.41) |  |
| Antidiabetics |  |  | 0.531 |  | 0.803 |  | 0.708 |  | 0.803 |  | 0.427 |  | 0.739 |  | 0.901 |
| Yes | 7 | 1.26 ± 1.13 |  | 0.76  (0.14, 2.24) |  | 0.36  (0.22, 1.11) |  | 1.58 ± 1.03 |  | 0.68  (0.21, 2.02) |  | 1.94 ± 1.34 |  | 1.71 ± 1.49 |  |
| No | 27 | 1.01  (0.67, 2.11) |  | 0.36  (0.13, 2.61) |  | 0.38  (0.15, 0.78) |  | 1.44  (0.92, 1.95) |  | 0.82  (0.37, 1.53) |  | 1.32  (0.85, 3.07) |  | 1.36  (0.54, 2.79) |  |
| Hypertension |  |  | 0.298 |  | 0.969 |  | 0.939 |  | 0.701 |  | 0.316 |  | 0.759 |  | 0.939 |
| Yes | 25 | 1.48 ± 1.03 |  | 0.49  (0.14, 1.81) |  | 0.39  (0.17, 0.68) |  | 1.44  (0.97, 1.89) |  | 0.80  (0.43, 1.72) |  | 1.51  (0.92, 2.88) |  | 1.01  (0.51, 3.03) |  |
| No | 9 | 0.82  (0.67, 1.21) |  | 0.27  (0.14, 3.01) |  | 0.51 ± 0.38 |  | 0.95  (0.88, 2.34) |  | 0.73 ± 0.57 |  | 1.85 ± 1.40 |  | 1.53 ± 1.05 |  |
| Diabetes |  |  | 0.531 |  | 0.803 |  | 0.708 |  | 0.803 |  | 0.427 |  | 0.739 |  | 0.901 |
| Yes | 7 | 1.26 ± 1.13 |  | 0.76  (0.14, 2.24) |  | 0.36  (0.22, 1.11) |  | 1.58 ± 1.03 |  | 0.68  (0.21, 2.02) |  | 1.94 ± 1.34 |  | 1.71 ± 1.49 |  |
| No | 27 | 1.01  (0.67, 2.11) |  | 0.36  (0.13, 2.61) |  | 0.38  (0.15, 0.78) |  | 1.44  (0.92, 1.95) |  | 0.82  (0.37, 1.53) |  | 1.32  (0.85, 3.07) |  | 1.36  (0.54, 2.79) |  |
| Dyslipidemia |  |  | 0.201 |  | 0.403 |  | 0.468 |  | 0.612 |  | 0.971 |  | 0.176 |  | 0.663 |
| Yes | 23 | 0.96  (0.59, 1.70) |  | 0.49  (0.13, 1.00) |  | 0.38  (0.19, 0.85) |  | 1.44  (0.90, 2.21) |  | 0.82  (0.30, 1.53) |  | 1.32  (0.84, 2.68) |  | 1.36  (0.56, 2.80) |  |
| No | 11 | 1.75 ± 1.24 |  | 0.33  (0.14, 3.45) |  | 0.38 ± 0.27 |  | 1.29  (0.93, 1.94) |  | 0.68  (0.48, 1.53) |  | 1.79  (1.32, 4.59) |  | 1.12  (0.35, 2.34) |  |

Note: ACEIs: angiotensin-converting enzyme inhibitors; CCBs: calcium channel blockers; *: *p* < 0.05.
